# Metabolic and catecholamine response to sympathetic stimulation in early-treated adult male patients with phenylketonuria

**DOI:** 10.1007/s42000-020-00176-z

**Published:** 2020-01-28

**Authors:** Csaba Sumanszki, Krisztian Kovacs, Gellert Balazs Karvaly, Erika Kiss, Erika Simon, Attila Patocs, Miklos Toth, Zsolt Komka, Peter Reismann

**Affiliations:** 1 2nd Department of Internal Medicine, Faculty of Medicine, Semmelweis University, Szentkirályi u. 46, Budapest, 1088 Hungary; 2 Department of Laboratory Medicine, Semmelweis University, Budapest, Hungary; 3 1st Department of Pediatrics, Semmelweis University, Budapest, Hungary; 4 Molecular Medicine Research Group, Hungarian Academy of Sciences and Semmelweis University, Budapest, Hungary; 5 “Lendület” Hereditary Endocrine Tumours Research Group, Hungarian Academy of Sciences and Semmelweis University, Budapest, Hungary; 6 Department of Health Sciences and Sport Medicine, University of Physical Education, Budapest, Hungary

**Keywords:** PKU, Epinephrine, Norepinephrine, Phenylalanine, Sympathetic stress test

## Abstract

**Purpose:**

Defective function of phenylalanine hydroxylase in phenylketonuria (PKU) results in the accumulation of phenylalanine (Phe) and the reduction of tyrosine (Tyr) in the blood, interfering in the normal development and function of organs and tissues in the body. Tyr is the precursor of catecholamines, secreted in response to stress by the adrenal medulla and paraganglia. The aim of this study was to evaluate plasma catecholamine and amino acid response to an escalating series of sympathetic stress tests in PKU patients.

**Methods:**

Twelve males with classical PKU (aged 18–41 years) and ten healthy male controls were included in this study. The subjects were exposed to three different sympathetic stress stimulations: cold pressor, isometric handgrip, and peak treadmill tests to exhaustion. Physiological, metabolic, and hormonal changes were determined.

**Results:**

Aerobic capacity (VO_2max_) was significantly lower in the PKU group (*p* = 0.018); however, relative VO_2max_ was similar in the two groups during the spiroergometric test. No significant differences in norepinephrine or in epinephrine response were found between the two groups during the different stimulation tests. Blood Phe increased significantly in the PKU group compared with controls (*p* = 0.027) during the spiroergometric test, while Tyr levels remained stable in both groups.

**Conclusion:**

PKU itself might not influence stress-induced catecholamine changes. Only strenuous exercise increased blood Phe levels in PKU subjects.

## Introduction

Phenylketonuria (PKU, OMIM 261600) is an autosomal recessive metabolic disease caused by mutations of the phenylalanine hydroxylase (PAH) gene (*612349), which leads to the incomplete hepatic conversion of the essential amino acid phenylalanine (Phe) to tyrosine (Tyr). The toxic accumulation of Phe along with low concentrations of Tyr and other neurotransmitter precursors in the brain is known to be responsible for the severe mental impairment and other major neurological and psychological complications (seizures and behavioral problems) in PKU [1]. To prevent these major sequelae, a lifelong, natural low-protein diet is initiated after a positive diagnosis during newborn screening in order to achieve and maintain secure blood Phe concentrations. Tyr supplementation alone does not correct the phenotype. Daily consumption of Phe-free amino acid supplements (AAS) completes the low-protein diet with the necessary amino acids, vitamins, minerals, and trace elements [2].

Tyr is needed for the biosynthesis of catecholamines (epinephrine, norepinephrine, and dopamine), thyroxin hormone, and melanin pigment [3]. Catecholamines are synthesized from Tyr in the central nervous system (CNS) and in the periphery by the adrenal medulla and paraganglia. Tyrosine hydroxylase (TYH) is the rate-limiting enzyme of the entire catecholamine synthetic pathway, transforming Tyr into levodopa (L-DOPA). L-DOPA, in turn, is transformed into dopamine (DA) in the presence of the DOPA decarboxylase (AAAD) enzyme. DA is transformed into norepinephrine (NE) in the presence of the dopamine ß-hydroxylase (DBH). Norepinephrine is eventually transformed into epinephrine (E) in the presence of phenylethanolamine N-methyltransferase (PNMT), an enzyme found primarily in the adrenal medulla [4].

Catecholamines function as monoamine neurotransmitters in the CNS and as adaptive stress hormones in the periphery. Sympathetic stimulation induces increased activities of both TYH and AAAD, creating a surge in catecholamines, mainly E and NE, from the adrenal medulla and paraganglions. The catecholamines in the bloodstream interact with the adrenergic receptors located in cell membranes, thus increasing blood pressure and cardiac output, relaxing bronchial, intestinal, and many other smooth muscles, and causing mydriasis and metabolic changes that increase levels of blood glucose and free fatty acids. [5].

Deficiencies of the monoamine neurotransmitters, particularly DA and serotonin, have been found to have major roles in the development of neurophysiological symptoms in PKU patients with elevated Phe levels [6]. Two possible hypotheses have been developed regarding the impairment of catecholamine metabolism in PKU. The first hypothesis suggests that high blood Phe levels competitively inhibit transport of Tyr and tryptophan (Trp), amino acid precursors of catecholamines and serotonin, respectively, across the blood–brain barrier [7]. The second hypothesis proposes a phenylalanine-mediated competitive inhibition of TYH and TRP, the rate-limiting enzymes in catecholamine and serotonin synthesis [8]. While the first mechanism is specific to the CNS, the second could have an effect on peripheral catecholamine metabolism. Few studies have investigated plasma catecholamine levels in PKU patients. A study conducted in a pediatric population found significantly lower plasma catecholamine levels in non-adherent PKU patients compared with compliant PKU patients and controls [9]. However, a dynamic study conducted in a mixed age and gender population found no significant difference in plasma catecholamine levels of PKU patients compared with controls [10].

The aim of the present study was to evaluate the catecholaminergic, metabolic, and physiological responses to mild–moderate and intense sympathetic stimulation. Our working hypothesis was that the physiological and catecholaminergic response would be altered by the elevated Phe levels in PKU subjects.

## Methods

### Patients

In this monocentric study, 12, early-treated PKU (ETPKU) male patients (aged 18–41 years) and 10 healthy controls (aged 24–27) were enrolled between December 2016 and December 2017. Inclusion criteria were (a) classical PKU diagnosis made during neonatal screening, (b) early and continuous treatment initiated after diagnosis, (c) older than 18 years of age, and (d) good compliance regarding regular checkups at the adult metabolic center.

The study was performed at the Semmelweis University, Budapest, Hungary, and at the University of Physical Education, Budapest, Hungary, on two separate days between 7 am and 2 pm. A light breakfast was permitted 1 h before the tests. Smoking and drinking beverages containing caffeine or alcohol were prohibited for 12 h before the tests. On both days, after admission, a heparin lock was placed in an antecubital vein of the subjects for blood sampling. Subjects were then asked to rest in a supine position for 30 min, in a quiet dimly lit room. After this resting period, basal heart rate and blood pressure were measured and resting blood samples were collected. The first day included two mild sympathetic stimuli: the cold pressor test (CPT) and the isometric handgrip test (HGT). On a separate day, an intense sympathetic test, namely, the peak treadmill test to exhaustion, was performed.

### Cold pressor test

The subjects were asked to immerse their non-dominant hand into a bucket containing ice water (2 °C) for as long as he could tolerate the cold (maximum of 2 min). Heart rate was monitored continuously and blood pressure before the completion of the test. When the subject pulled his hand from the ice water, a blood sample for catecholamines and amino acids measurement was collected.

### Isometric handgrip test

The subjects were asked to hold the handgrip dynamometer, Jamar Hydraulic Hand Dynamometer-200 lb. (Patterson Medical, Warrenville, IL, USA), in the right (or dominant) hand. Maximum effort was determined by compression of the handles as intensely as possible for a few seconds, three times. The average of the three compressions was used to determine the maximal isometric tension (cold-pressor). The subjects were then asked to perform the isometric handgrip exercise at 30% of *T*_max_ for 3 min. Heart rate was monitored continuously and blood pressure was measured before the completion of the exercise. After the termination of the test, blood samples for catecholamines and amino acid measurement were collected.

### Peak treadmill test to exhaustion

In order to achieve maximum physical stress, each subject participated in a peak treadmill test to exhaustion at the University of Physical Education. An Ergo-Fit Cardio Line 4000 TRAC (EuroMedix, Leuven, Belgium) treadmill ergometer was used with a Cardiovit AT-104 ECG recorder (Schiller Medizintechnik GmbH, Ottobrun, Germany), in conjunction with a PowerCube (Ganshorn Medizin Electronic GmbH, Niederlauer, Germany) O_2_ and carbon dioxide (CO_2_) gas analyser. The system was calibrated before each measurement. The classic “vita maxima type” criteria was used to evaluate VO_2max_ (reaching the plateau in oxygen uptake, maximal respiratory exchange ratio higher than 1.1, and 90% of age-predicted HRmax) [11]. Tests were terminated when the subjects reached the maximum oxygen uptake criteria or had subjective complaints (fatigue, pain, or dizziness). Modified Bruce protocol with constant running speed and increments of 1.5% every minute was applied after 4 min of the warm-up period (2 min 4 km/h and 2 min 5 km/h speed, 0% tilt).

### Lactate measurement

Blood lactate concentration was determined by using a blood lactate meter supplied by Nova Biomedical (Waltham, MA, USA). The blood for the measurement was obtained from the ear lobe before and immediately after finishing the working load and 5 min later.

### Body composition measurement

Direct segmental multi-frequency BIA measurements were taken using BIA InBody 720 (BioSpace Co., Seoul, Korea).

### Blood amino acid measurement

All blood samples were drawn from the antecubital vein under standardized conditions. Phe and Tyr levels were measured by API2000 LC/MS/MS (Perkin-Elmer Sciex, Ontario, Canada) at the 1st Department of Pediatrics, Semmelweis University, Budapest.

### Catecholamine level measurement

E and NE were assayed in plasma using liquid chromatography coupled with amperometric detection. The chromatographic system consisted of a JASCO PU-4180 isocratic pump and an AS-4050 autosampler equipped with a TC-4000-1 cooling unit. An ANTEC DECADE Lite electrochemical detector with a VT-03 flow cell was employed for detection (ABL&E-JASCO Magyarország Kft, Budapest, Hungary).

The analysis was performed using a Chromsystems™ reagent kit for HPLC analysis of catecholamines in plasma (No. 5000) and a Chromsystems HPLC column, equilibrated, with test chromatogram, for catecholamines in plasma (No. 5100, ABL&E-JASCO Magyarország Kft, Budapest, Hungary). Briefly, sample preparation consisted of diluting 1.0 mL plasma samples with a buffer and adding the internal standard provided with the reagent kit, followed by extraction onto an adsorbent, washing, and elution. An 80 μL sample was injected into the chromatographic system. The stationary phase was kept at ambient temperature and the mobile phase flow rate was 0.9 mL/min. For detection, the working electrode potential was set at 550 mV, the cell current range at 50 nA, and the filter rate at 0.2 Hz. Single-level calibration was performed using a lyophilized calibrator sample. Analyte levels in the calibrator (Chromsystems Plasma Calibration Standard, Catecholamines in Plasma, No. 5009) slightly varied from lot to lot (< 5%), with median norepinephrine and epinephrine levels of 1132 and 290 pg/ml, respectively. Bi-level controls (Chromsystems Endocrine Plasma Control, No 0010/0020) were run parallel with the study samples.

Peak heights were evaluated for quantitation. The ratio of the peak height of each analyte and that of the internal standard was used for calculations.

### Statistical analysis

Statistical analysis was performed using statistical package SPSS version 23 (IBM Corp. in Armonk, NY, USA). Results were reported as median and 25th and 75th percentiles. Because of the small sample size, the Mann–Whitney U test was used to test for differences between subgroups. *P*-values < 0.05 were considered significant.

## Results

Baseline characteristics of the 12 PKU subjects and 10 control subjects are listed in Table 1. No significant difference in age, BMI, and fractioned mass was observed. Resting blood pressure and heart rate were comparable between the two groups. As expected, blood Phe was significantly higher in the PKU group compared with the control group on both days of the tests (*p* < 0.001 and *p* < 0.001, respectively), while Tyr did not differ significantly between the two groups. Baseline catecholamine levels after 30 min of resting where similar in the two groups (Table 1).

**Table 1:** Anthropometric data and baseline laboratory parameters

	**Control subjects (*n*** **= 10)**	**PKU subjects (*n*** **= 12)**	*p*
**Age (years)**	26 (24.7–27)	26 (20.2–36.7)	0.958
**Height (cm)**	181 (175.6–184.3)	179.3 (174.6–183.5)	0.615
**Weight (kg)**	74.7 (66.4–87.7)	69.5 (65.8–80)	0.221
**BMI (kg/m**^**2**^**)**	22.5 (21.6–26.7)	23.15(20.3–25.2)	0.710
**Body fat (%)**	16.6 (13.8–20.9)	16.1 (11.9–25.5)	0.909
**Body muscle (%)**	47.3 (45.3–48.5)	47.5 (42.1–49.3)	0.497
**Body bone (%)**	4.6 (4.4–4.8)	4.5 (4–4.8)	0.328
**Residual (%)**	31.3 (29.1–32.5)	31.3 (29.1–32.5)	0.949
**Resting HR (beats/min)**	68.5 (57.2–76)	63 (56.2–73.7)	0.685
**Resting systolic BP (mmHg)**	123.5 (118.5–126.5)	119.5 (110.5–123.8)	0.178
**Resting diastolic BP (mmHg)**	69.5 (64.5–74.2)	66.5 (64.2–70)	0.328
**Phe (μmol/L)**	43.4 (39.7–58.7)	562.1 (361.8–727.3)	***< 0.001***
**Tyr (**μmol**/L)**	47.4 (42.0–55.7)	39.4 (32.5–79.9)	0.719
**NE (pg/ml)**	344.4 (191.1–414.5)	224.1 (201–265)	0.203
**E (pg/ml)**	27.4 (21.7–38.9)	34.2 (30.3–41.1)	0.274

Values are presented as median (25th and 75th percentiles)

*Abbreviations*: *HR* heart rate, *BP* blood pressure, *Phe* phenylalanine, *Tyr* tyrosine, *NE* norepinephrine, *E* epinephrine

Differences between the two groups were tested using the Mann–Whitney U test.

### Cold pressor test

Changes in physiological response, heart rate, and blood pressure during the cold pressor test were similar in the two groups. No significant changes in metabolic parameters or Phe and Tyr levels were observed in the PKU group compared with the control group. Changes in catecholamine levels were comparable between the two groups (Table 2).

**Table 2:** Blood pressure, heart rate, and laboratory parameters during cold pressor test

	**Control subjects (n = 10)**	**PKU subjects (n = 12)**	*p*
**∆ HR (beats/min)**	25 (17–32)	21.5 (14–30.5)	0.473
**∆ Systolic BP (mmHg)**	16 (5.5–23)	16.5 (14–24.7)	0.472
**∆ Diastolic BP (mmHg)**	12 (4.5–20)	15.5 (8.5–17.7)	0.638
**∆ Phe (**μmol**/L)**	0.8 (− 2.7–2.0)	7.3 (− 12.9–48.7)	0.764
**∆ Tyr (μmol/L)**	0.4 (− 5.5–3.1)	3.2(− 1.6–11.6)	0.211
**∆ NE (pg/ml)**	61.6 (22.3–190.5)	81.9 (31.8–103.4)	0.915
**∆ E (pg/ml)**	10.9 (7.4–45.4)	17.9 (8.7–53.8)	0.680

Values are presented as median (25th and 75th percentiles)

*Abbreviations*: *∆* change from resting, *HR* heart rate, *BP* blood pressure, *Phe* phenylalanine, *Tyr* tyrosine, *NE* norepinephrine, *E* epinephrine

Differences between the two groups were tested using the Mann–Whitney U test.

### Isometric handgrip test

Changes in heart rate and blood pressure from resting values were comparable between the two groups during the isometric handgrip test. Changes in Phe levels were higher in the PKU compared with the control group, although statistically not significant. Changes in Tyr levels were comparable in both groups. Changes in catecholamine levels were not significantly different in the two groups (Table 3).

**Table 3:** Blood pressure, heart rate, and laboratory parameters during isometric handgrip test

	**Control subjects (n = 10)**	**PKU subjects (n = 12)**	*p*
**∆ HR (beats/min)**	20 (15–24)	16 (12.2–25.2)	0.491
**∆ Systolic BP (mmHg)**	23.5 (7.7–48.2)	36.5 (19–43.5)	0.261
**∆ Diastolic BP (mmHg)**	18 (10–40)	13.5 (4.7–19.5)	0.371
**∆ Phe (**μmol**/L)**	2.7 (− 1.3 – 5.9)	11.1 (−33.5–37)	0.242
**∆ Tyr (μmol/L)**	0.4 (− 5.5–3.1)	− 1.4 (− 8.2–0.9)	0.645
**∆ NE (pg/ml)**	61.2 (34.0–209.5)	40.6 (17.7–98.8)	0.999
**∆ E (pg/ml)**	14.5 (5.4–53.5)	14.2 (4.9–27.6)	0.541

Values are presented as median (25th and 75th percentiles)

*Abbreviations*: *∆* change from resting, *HR* heart rate, *BP* blood pressure, *Phe* phenylalanine, *Tyr* Tyrosine, *NE* norepinephrine, *E* epinephrine

Differences between the two groups were tested using the Mann–Whitney U test.

### Peak treadmill test to exhaustion

All the subjects completed the treadmill test. No significant difference in either the aerobe (aerobic) or anaerobe (anaerobic) duration of exercise was observed in the PKU subjects compared with controls. Maximal heart rate was significantly higher in the control group compared with the PKU group (*p* = 0.037). No significant differences were observed in blood pressure changes between the two groups. The relative aerobic capacity expressed as VO_2max_ was significantly lower in the PKU group compared with the controls (*p* = 0.018); however, relative VO_2max_ was similar in the two groups. Cumulative workload measured in watt was significantly higher in the control group compared with the PKU group (*p* = 0.002). Although PKU subjects had lower lactate levels after the exercise test and at restitution, they were not significantly different compared with those of the control group. Phe levels increased by 4.9% in PKU subjects and were significantly higher compare with those of the control group (*p* = 0.027). Catecholamine response upon maximal workload was comparable between the two groups (Table 4).

**Table 4:** Exercise and laboratory parameters before and after the treadmill test

	**Control subject (n = 10)**	**PKU subjects (n = 12)**	*p*
**Aerobic time (sec)**	556 (480.3–683.5)	498 (285–664)	0.883
**Anaerobic time (sec)**	177 (129.8–204.3)	154 (140.8–271.8)	0.247
**HR resting**	71.5 (65.2–73.2)	67 (64.2–73.7)	0.228
**Max HR (beats/min)**	197.5 (191.8–203.3)	188.5 (185.5–199)	***0.037***
**Systolic BP resting**	126 (112–134.5)	120.5 (109.3–126)	0.153
**Max systolic BP (mmHg)**	172 (165–191.8	168 (162–172)	0.426
**∆ Systolic BP (mmHg)**	46.5 (36.2–65.2)	51 (46–57)	0.657
**Diastolic Bp resting**	71.5 (66–83.5)	72.5 (67.5–83.2)	0.708
**Max diastolic BP (mmHg)**	89 (84.7–97.2)	90 (73–100)	0.974
**∆ Diastolic BP (mmHg)**	17.5 (6–26.2)	20 (6–35)	0.703
**VO**_**2max**_ **(ml/min)**	3970 (3758–4108)	3080 (2813–3768)	***0.004***
**Relative VO**_**2max**_ **(ml/kg/min)**	48.5 (46.5–59)	45 (36.3–52.8)	0.174
**Cumulative workload (MET)**	13.8 (13.2–16.8)	12.8 (10.4–15.1)	0.185
**Cumulative workload (Watt)**	336.5 (316–380.8)	253.5 (207–303)	***0.003***
**Max RQ**	1.08 (1.0–1.1)	1.1 (1.08–1.1)	0.070
**Resting lactate (mM)**	1.4 (1.0–1.7)	1.2 (0.9–1.7)	0.285
**Max lactate (mM)**	10.9 (9.3–11.8)	9.3 (6.9–10.8)	0.140
**Restitution 5′ lactate (mM)**	11.3 (10.4–13.4)	9.1 (8.0–11.9)	0.093
**Phe resting (**μmol**/L)**	43.7 (37.2–57.9)	542.3 (467.9–709.4)	***<0.001***
**∆ Phe exercise (**μmol**/L)**	1.8 (− 3.3–7.0)	26.7 (2.8–65.1)	***0.027***
**∆ Phe restitution (μmol/L)**	− 7.9 (− 20.4–− 2.9)	− 2.5 (− 50.3–22.5)	0.882
**Tyr resting (μmol/L)**	46.6 (39.2–54.8)	37.6 (29.6–49.6)	0.199
**∆ Tyr exercise (μmol/L)**	− 1.45 (− 2.8–0.8)	− 3.5 (− 5.2–2.0)	0.899
**∆ Tyr restitution (μmol/L)**	− 4.5 (− 6.5–− 1.7)	− 1 (− 5.7–2.5)	0.111
**NE baseline (pg/ml)**	300.5 (250–442.5)	287.5 (190.8–404.5)	0.614
**NE exercise (pg/ml)**	5008 (3482–11,689)	3726 (2401–4760)	0.140
**∆ NE (pg/ml)**	4745 (3175–11,159)	3448 (2151–4324)	0.122
**E baseline (pg/ml)**	54.5 (44.5–80.7)	66 (51.5–74)	0.662
**E exercise (pg/ml)**	507 (280.5–1327)	457 (317.8–1306)	0.956
**∆ E (pg/ml)**	428.5 (164–1170)	386 (250.3–1238)	0.771

Values are presented as median (25th and 75th percentiles)

*Abbreviations*: *∆* change from resting, *HR* heart rate, *BP* blood pressure, *VO*_*2max*_ maximal oxygen consumption, *RQ* respiratory quotient, *Phe* phenylalanine, *Tyr* Tyrosine, *NE* norepinephrine, *E* epinephrine

Differences between the two groups were tested by the Mann–Whitney U test.

## Discussion

To our knowledge, this is the first study that has compared the metabolic and catecholaminergic response to three levels of stress in adult early-treated PKU subjects with those of healthy control subjects. Participant selection was limited to adult male participants, as it has previously been shown that gender and age can influence catecholaminergic response to stress [12, 13].

Adaptive response to stress is vital, since any dysfunction in stress reaction can substantially influence health outcome. The main stress hormones besides cortisol are the catecholamines, derived from Tyr. In non-treated or non-adherent PKU patients, Tyr levels are low and Phe levels are high [14, 15]. Both the low availability of Tyr and the potential suppressive effect of high Phe levels on enzymatic function can significantly influence catecholamine production [16]. A natural protein-restricted diet lowers blood Phe, while the regular consumption of AAS can restore blood Tyr levels, though with a large daily fluctuation [17]. It is not at present known, whether a modest elevation of Phe and a great fluctuation of Tyr—as seen in ETPKU patients—can alter catecholamine metabolism. To study the stress reaction of patients, three different types of stress were introduced. The cold pressor test and the isometric handgrip test evoke modest stress response, while the treadmill ergometric test provokes pronounced stress reaction.

Resting catecholamine levels were comparable between the two groups. Similarly, Mazzola et al. also found no significant difference in basal catecholamine levels between PKU subjects and healthy controls [10]. Therefore, it is questionable whether plasma catecholamine levels are only affected in children with PKU [18, 19]. The catecholamine surge was comparable during the modest stress challenges, like the CPT and HGT test. Correspondingly, no significant difference in blood pressure was observed between PKU and control subjects. The exhaustive nature of the maximal workload test was evident from the mean post-exercise blood lactate levels. We observed slightly worse performance of PKU patients during the treadmill test, demonstrated by significantly lower VO_2max_ and cumulative workload; nonetheless, performance adjusted to weight, represented by relative VO_2max_, was not significantly different between the two groups. The difference in performance might be influenced by the different nutrition, but this should be further studied**.**

Catecholamine levels measured immediately after completion of the exercise were not significantly different from the control groups. These findings suggest similar catecholamine response to all three stress situations of PKU subjects compared with control subjects.

The observed changes in Phe and Tyr levels in the PKU and control groups during the treadmill test were in line with previous observations regarding plasma amino acid changes after exercise [20]. During high-intensity exercise, there is a marked increase in plasma amino acids due to protein catabolism, followed by protein synthesis in the resting period [20, 21]. The difference in Phe level changes between the two groups can be explained by PKU subjects’ impaired ability to metabolize the Phe accumulated from protein degradation during exercise. Nonetheless, the increase of 4.9% in Phe levels in the PKU group after strenuous exercise was negligible compared with postprandial changes of Phe levels of PKU subjects [22]. Tyr levels were comparable and remained stable during the exercise tests in both groups. A recent study comparing blood Phe and Tyr levels before and after 1 h of ergometric endurance exercise of PKU subjects found no difference in Phe levels and a slight decrease in Tyr levels [23]. Another study similarly showed no significant increase in Phe levels after submaximal exercise [10]. The discrepancy between the previous studies and our findings could be due to the different intervals of blood sample collection and exercise intensity.

Our study has some limitations worth pointing out. Firstly, the number of control participants was small, and age matching of the PKU and control group was not performed. Secondly, plasma dopamine measurement was not available due to technical difficulties.

## Conclusion

Stress response during an escalating series of sympathetic tests resulted in similar hormonal changes between PKU and control subjects, suggesting adequate catecholamine production.

Modest elevation of Phe seems not to influence Tyr-dependent catecholamine metabolism.

High-intensity exercise appears to be safe in PKU subjects, since no dramatic increase in blood Phe was recorded.
